# Redox-Responsive Crosslinked Mixed Micelles for Controllable Release of Caffeic Acid Phenethyl Ester

**DOI:** 10.3390/pharmaceutics14030679

**Published:** 2022-03-20

**Authors:** Katya Kamenova, Georgy Grancharov, Vasilena Kortenova, Petar D. Petrov

**Affiliations:** Institute of Polymers, Bulgarian Academy of Sciences, Akad. G. Bonchev St., Block 103-A, 1113 Sofia, Bulgaria; kkamenova@polymer.bas.bg (K.K.); granchar@polymer.bas.bg (G.G.); vasilenakortenova@gmail.com (V.K.)

**Keywords:** block copolymers, co-assembly, redox-responsive mixed micelles, nanocarriers, caffeic acid phenethyl ester

## Abstract

We report the elaboration of redox-responsive functional micellar nanocarriers designed for triggered release of caffeic acid phenethyl ester (CAPE) in cancer therapy. Three-layered micelles, comprising a poly(ε-caprolactone) (PCL) core, a middle poly(acrylic acid)/poly(ethylene oxide) (PAA/PEO) layer and a PEO outer corona, were prepared by co-assembly of PEO_113_-*b*-PCL_35_-*b*-PEO_113_ and PAA_13_-*b*-PCL_35_-*b*-PAA_13_ amphiphilic triblock copolymers in aqueous media. The preformed micelles were loaded with CAPE via hydrophobic interactions between the drug molecules and PCL core, and subsequently crosslinked by reaction of carboxyl groups from PAA and a disulfide crosslinking agent. The reaction of crosslinking took place in the middle layer of the nanocarriers without changing the encapsulation efficiency (EE~90%) of the system. The crosslinked polymeric micelles (CPMs) exhibited superior structural stability and did not release CAPE in phosphate buffer (pH 7.4). However, in weak acidic media and in the presence of 10 mM reducing agent (dithiothreitol, DTT), the payload was released at a high rate from CPMs due to the breakup of disulfide linkages. The physicochemical properties of the nanocarriers were investigated by dynamic and electrophoretic light scattering (DLS and ELS) and atomic force microscopy (AFM). The rapid release of CAPE under intracellular-like conditions and the lack of premature drug release in media resembling the blood stream (neutral pH) make the developed CPMs a promising candidate for controllable drug release in the microenvironment of tumors.

## 1. Introduction

Nanocarriers play an important role in various strategies aimed at improving the efficiency of antineoplastic agents and overcoming the existing limitations of conventional chemotherapy. Nanocarrier-based formulations have demonstrated good drug protection against enzymatic degradation, minor side effects, high cell uptake, targeted delivery to a specific area (e.g., solid tumor), and controlled/triggered drug release [1]. Systems that combine such functions can significantly improve the effectiveness of cancer treatment. Polymeric micelles, comprising a hydrophobic core and a hydrophilic shell, have been considered one of the most promising nanocarriers of hydrophobic antineoplastic drugs [2]. Polymeric micelles are aggregates constructed by self-assembly of amphiphilic copolymers in an aqueous environment. These amphiphilic copolymers can be properly designed in terms of macromolecular architecture, hydrophilic/lipophilic balance, functionality, molar mass, etc., for achieving favouable micellar dimensions, high drug loading capacity, good in vivo structural stability, longevity, targeting properties and low toxicity, among others [3]. For example, micelles comprising a poly(ethylene oxide) shell have been found to circulate in the blood stream for extended periods because they evade the mononuclear phagocytic system (“stealth” effect) and at the same time are large enough to avoid a fast renal clearance [4]. The prolonged circulation of carriers in the blood provides opportunities to maintain a therapeutic dose for a given time (hours to days) or to deliver cargo to a target zone. The release of drug from nanocarriers mainly follows the physicochemical properties of the materials, and, for conventional polymeric micelles, is generally controlled by diffusion mechanism and degradation [5]. By using “smart” micellar systems, one can adjust the release of drug through the interaction between nanomaterials and changes in their environment (i.e., pH, redox potential, enzyme concentration, etc.). Such carriers are expected to accumulate in the target site using a passive or active targeting mechanism, and then to quickly release the drug upon specific stimuli (controllable release) [6]. This approach can increase the therapeutic efficacy and diminish systemic side effects [7]. Therefore, upgrading existing systems and the development of novel stimuli-sensitive micellar carriers is of great importance for advanced chemotherapy.

Since tumor cells have different microenvironments compared to healthy ones, this can be exploited to activate drug release from the micelles. Hence, various intracellular stimuli, including reactive oxygen species, endosomal/lysosomal pH (pH 6.5–4.5), redox potential, over-expressed enzymes, etc., have been studied for controlling the action of micelles inside cells [3,8]. For instance, the pH in tumor cells is lower (∼6.5) than the normal tissue cells (∼7.4) and tends to fall in intracellular organelles, such as lysosomes (∼5) [9]. pH-gradient offers an opportunity for release of therapeutic payloads from pH-sensitive polymeric nanocarriers upon a change in the pH of their microenvironment. Kataoka et al. developed pH-sensitive micellar nanocarriers from poly(ethylene glycol)-b-poly(aspartate) diblock copolymer for co-delivery of staurosporine, a cancer stem cell inhibitor, and the anticancer agent epirubicin [10]. Epirubicin was linked to the copolymer through acid-labile hydrazone bonds, which allowed it to be triggered and to have a coordinated release at endosomal pH.

In addition to pH changes, intracellular cleavage of covalent bonds for selective drug release from polymeric micelles could be attained through either oxidation or reduction reactions. The fact that the concentration of glutathione (GSH) in tumor cells is significantly (>100-fold) higher than its concentration (2–10 μM) in plasma and the capability of GSH to cleave disulfide bonds has motivated numerous researchers to fabricate polymeric micelles containing SS-bonds for triggered release of antineoplastic drugs [8]. Generally, such micellar carriers are designed to hold the cargo under normal conditions and release it upon destabilization, due to the pronounced cleavage of disulfide bonds by GSH inside the cancer cells. The disulfide linkages have been incorporated in the polymeric micelles via crosslinking segments of the micellar core, shell, or the core–shell interface, or in the main polymer chain to induce the cleavage of the block copolymer and micelle disruption [3]. The covalent crosslinking of micelles is considered more beneficial as it can prevents undesired disaggregation and drug loss [11,12]. Li and co-workers synthesized thiolated linear-dendritic polymers from polyethylene glycol (PEG) and a dendritic cluster of cholic acids [13]. Reversibly cross-linked micelles were then prepared via self-assembly of the thiolated telodendrimers and subsequent oxidization of the thiol groups to disulfide bonds in the core of the micelles. However, crosslinking of the micellar core reduced the drug loading capacity of the carriers. The crosslinking of the micellar shell can bypass such a problem, but, at a very high crosslinking density, it can alter the properties of the hydrophilic polymer and lower the colloid stability and longevity of the carriers. On the other hand, when a network is loose, the crosslinked shell cannot stop undesired leakage of the drug [14]. Hence, another valuable strategy used to preserve the favorable characteristics of both the inner core and outer hydrated layer is based on shell crosslinking of micellar aggregates comprising a core–shell–corona structure. Koo et al. prepared disulfide-crosslinked micelles from an amphiphilic ABC triblock terpolymer of poly(ethylene glycol)-*b*-poly(L-lysine)-*b*-poly(L-phenylalanine) (PEG-*b*-PLys-*b*-PPha) [15]. This terpolymer formed aggregates of well-defined and distinct domains, consisting of a hydrophobic PPha core, a PLys middle shell, containing reactive primary amines, and a hydrated PEG outer corona. The crosslinks in the middle shells not only enhanced the micellar stability against micelle-destabilization, but also avoided drug release in extracellular environments. Thereafter, different reversible cross-linked polymeric micelles intended for intracellular delivery of anticancer agents, such as doxorubicin [16,17,18], camptothecin [19], and curcumin [20], have been developed. It should be noted that the synthesis of ABC triblock terpolymers, comprising specific reactive groups in the middle block, is not an easy task and involves many different reaction and purification steps. Alternatively, functional multilayered micelles can be formed by cooperative self-assembly (co-assembly) of two (or more) block copolymers having different intrinsic properties [21]. This approach is considered more flexible and easier to implement than the self-assembly of a single multiblock co- or terpolymer.

Caffeic acid phenethyl ester is one of the most intriguing bioactive constituents of propolis, with recognized antimicrobial, antioxidant, and anticancer activities [22]. Due to the hydrophobic nature of CAPE, its solubility in body fluids and bioavailability is very low. This is the main obstacle for in vivo administration of CAPE and its use in emerging areas, such as tumor therapy. Recently, a few studies have reported the positive effects of micellar core–shell nanocarriers towards enhancing the solubility of CAPE in aqueous media [23,24,25,26]. The systems exhibited sustained release profiles, with or without burst release, depending on the nature of the core-forming polymers. Moreover, functional micellar nanocarriers for codelivery of CAPE and doxorubicin were successfully developed via co-assembly of PEO-*b*-PCL-*b*-PEO and PAA-*b*-PCL-*b*-PAA block copolymers in water [27]. Although entrapping CAPE into the hydrophobic PCL core resulted in delayed drug release, these systems could not realize a controllable, trigger-induced release of CAPE at a pathological site, such as a solid tumor.

The present work describes the elaboration of novel redox-responsive micellar carriers for intracellular delivery of CAPE. Three-layered mixed micelles, comprising a PCL-core, a PAA/PEO middle layer, and an outer PEO corona were prepared by co-assembly of PEO_113_-*b*-PCL_35_-*b*-PEO_113_ and PAA_13_-*b*-PCL_35_-*b*-PAA_13_ and then loaded with CAPE. Next, the middle layer of nanocarriers were crosslinked via reacting the carboxyl groups of PAA with cystamine dichloride. The effect of SS-group containing nanonetwork on the structural stability of micelles and premature/triggered release of CAPE at conditions resembling the plasma and tumor cells was assessed. We found that the obtained nanocarriers are extremely stable and do not release any detectable amount of CAPE in a phosphate buffer of pH 7.4, while having rapid drug release in weak acidic media, in the presence of the reducing agent dithiothreitol.

## 2. Materials and Methods

### 2.1. Materials

Poly(ε-caprolactone) diol (CAPA^®^ 2402, molar mass 4000 g mol^−1^, Solvay Chemicals Inc., Houston, TX, USA) and methoxy poly(ethylene glycol) (molar mass 5000 g mol^−1^, Sigma-Aldrich) were precipitated in cold methanol (−30 °C), filtered, and dried under vacuum at 40 °C overnight. Tert-butyl acrylate (*t*BA, Sigma-Aldrich, Darmstadt, Germany) was passed through a column of alumina to remove the inhibitor. Copper (I) bromide (CuBr, Sigma-Aldrich, Darmstadt, Germany), pentamethyldiethylenetriamine (PMDETA, Sigma-Aldrich), α-bromoisobutyryl bromide (Sigma-Aldrich), triethylamine (Fluka, Fisher Scientific, Loughborough, UK), 4-pentynoic acid (Acros, through Labimex, Sofia, Bulgaria), sodium azide (NaN_3_, Fluka), 4-dimethylaminopyridine (Sigma-Aldrich), N-(3-dimethylaminopropyl)-Nʹ-ethylcarbodiimide hydrochloride (EDC, Merck), CAPE (synthetic procedure described in [24]), *N*-hydroxysulfosuccinimide sodium salt (Sulfo-NHS, Sigma-Aldrich), DMF (Sigma-Aldrich), tetrahydrofuran (Sigma-Aldrich) Triton X-105 (Sigma-Aldrich), cystamine dichloride (Merk, Sofia, Bulgaria), dithiothreitol (Sigma-Aldrich), and ethanol (Acros) were used as received.

### 2.2. Analysis

A Zetasizer NanoBrook 90Plus PALS instrument, equipped with a 35 mW red diode laser (λ = 640 nm), was used to determine the hydrodynamic diameter and zeta-potential of micelles. The measurements were carried out at 37 °C and a scattering angle of 90° was used. The UV-vis absorption spectra of CAPE-containing samples were recorded on a Thermo Scientific UV-vis spectrophotometer, using quartz cells (path length 1 cm). Atomic force microscopy analyses were performed with a Bruker Dimension Icon microscope, under ambient conditions at a 1.00 Hz scan rate. A total of 2 μL of filtered micellar solution (1 gL^−1^) was dropped onto a freshly cleaned glass substrate and spin-coated at 2000 rpm. The measurements were conducted in tapping mode using using silicon cantilevers with resonance frequency of ca. 300 kHz and a spring constant of 40 N/m.

### 2.3. Synthesis of Block Copolymers

The synthesis of PEO_113_-*b*-PCL_35_-*b*-PEO_113_ and PAA_13_-*b*-PCL_35_-*b*-PAA_13_ was described in detail elsewhere [24,27]. In brief, PEO_113_-*b*-PCL_35_-*b*-PEO_113_ (M_n_^NMR^ = 14,000 gmol^−1^; M_w_/M_n_^GPC^ = 1.3) was obtained by “click” coupling reaction of a mono-alkyne terminated PEG_113_ (M_n_^NMR^ = 5100 gmol^−1^; M_w_/M_n_^GPC^ = 1.1) and a di-azide terminal PCL_35_-diol (M_n_^NMR^ = 4400 gmol^−1^; M_w_/M_n_^GPC^ = 1.36) with CuBr/PMDETA catalytic complex in N,N-dimethylformamide at 30 °C. PAA_13_-*b*-PCL_35_-*b*-PAA_13_ (M_n_^NMR^ = 5870 gmol^−1^) was obtained as follows: PtBA_13_-*b*-PCL_35_-*b*-PtBA_13_ (M_n_^NMR^ = 7330 gmol^−1^; M_w_/M_n_^GPC^ = 1.1) triblock copolymer was synthesized by ATRP of tBA, initiated by a bifunctional Br-PCL_13_-Br macroinitiator in acetone in the presence of PMDETA/CuBr catalyst complex at 50 °C for 48 h. Then, PtBA blocks were derivatized into PAA ones by hydrolysis with an excess of trifluoroacetic acid.

### 2.4. Preparation of Micelles

Three-layered micelles were prepared by co-assembly of PAA_13_-*b*-PCL_35_-*b*-PAA_13_ and PEO_113_-*b*-PCL_35_-*b*-PEO_113_ using the solvent evaporation method. The two copolymers (total mass = 10 mg) were blended at a molar ratio 1:3 in 5 mL THF, and the solution was added dropwise to 10 mL of PBS buffer (pH 7.4) at room temperature under stirring (700 min^−1^). After 30 min, the organic solvent was evaporated under vacuum at 40 °C to afford stable aqueous micellar solution with a concentration of 1 gL^−1^.

### 2.5. Preparation of Crosslinked Micelles

Crosslinked micelles were prepared by covalently crosslinking carboxyl groups of PAA segments with cystamine dihydrochloride. In a typical run, NHS (2.5 equiv. with respect to total number of carboxyl groups) and EDC HCl (2 equiv. to the total number of carboxyl groups) were added into 15 mL of micellar solution and stirred for 20 min at room temperature under an inert atmosphere. Then, cystamine dihydrochloride (0.5 equiv.) was added and the mixture was stirred for 24 h at 25 °C. Finally, CPMs were purified by dialysis (MWCO = 15,000) against PBS buffer for 24 h. A sample for structural stability test in organic solvent was prepared by dialyzing SPMs against THF for 72 h.

### 2.6. Drug Loading of Micellar Carriers

CAPE was loaded into the micelles prior the crosslinking procedure. CAPE (0.1 mg) was dissolved in 1 mL of ethanol and added dropwise to the aqueous solution of non-crosslinked micelles (10 mL). The sample was stirred for 30 min, and then the ethanol was removed under vacuum at 40 °C. The micellar solution was filtered (0.45 µm) and the filter was rinsed with ethanol to collect the free drug. The amount of non-loaded CAPE was determined by UV-vis measurements (λ = 330 nm), and the encapsulation efficiency (EE) was calculated from the following equation:

(1) $$EE\left(\%\right)=\frac{Total\;mass\;of\;CAPE-Mass\;of\;free\;CAPE}{Total\;mass\;of\;CAPE}\times 100$$

### 2.7. In Vitro Drug Release

The in vitro drug release of CAPE from the non-crosslinked and crosslinked micellar carriers was determined using the dialysis method. Solutions of drug-loaded micelles in PBS at pH 7.4 or 5.0, containing 0 or 10 mM DTT, were transferred into dialysis membrane bags (MWCO = 3500) and dialyzed against buffer (pH = 7.4 or 5.0), containing Triton-x100 (0.2 mM). The tests were conducted at 37 °C for a given time. Samples were taken from the external medium at fixed time interval and the concentration of the released CAPE was determined by UV-vis spectrophotometry (λ = 330 nm) using a calibration curve.

## 3. Results

### 3.1. Polymeric Micelles

**Figure 1:**
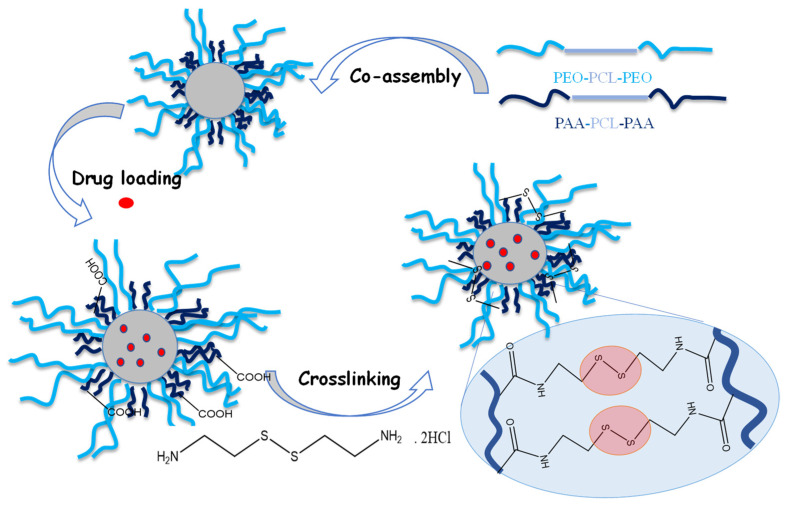
Schematic illustration of the preparation of redox-responsive crosslinked micellar nanocarriers based on PEO_113_-*b*-PCL_35_-*b*-PEO_113_ and PAA_13_-*b*-PCL_35_-*b*-PAA_13_ amphiphilic triblock copolymers.

CPMs containing CAPE were prepared as follows: The two triblock copolymers, PEO_113_-*b*-PCL_35_-*b*-PEO_113_ and PAA_13_-*b*-PCL_35_-*b*-PAA_13_, were blended (molar ratio 3:1) in THF, which is a common solvent for polymers. The polymer solution was added dropwise to PBS (pH 7.4) at 37 °C and allowed for 30 min to complete the co-assembly process (Figure 1, top). The organic solvent was evaporated and the obtained stable micellar solution (1 gL^−1^) was mixed with CAPE and dissolved in ethanol. The mass ratio micelles/CAPE was equal to 10:1. CAPE-loaded micelles were crosslinked by reacting the carboxyl groups of PAA segments with cystamine dihydrochloride, in the presence of EDC/NHS catalyst (Figure 1, bottom). The molar ratio of cystamine to the total carboxyl groups was set to 0.5; as we expected, one cystamine molecule reacted with two carboxyl groups.

**Figure 2:**
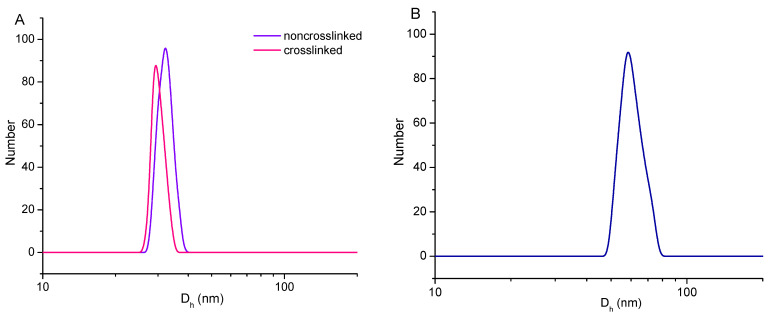
Size distribution plots of: (**A**) non-crosslinked and crosslinked micelles in PBS, and (**B**) crosslinked micelles in THF. Micelles were formed by co-assembly of PEO_113_-*b*-PCL_35_-*b*-PEO_113_ and PAA_13_-*b*-PCL_35_-*b*-PAA_13_ amphiphilic triblock copolymers at molar ratio 3:1.

The micellar aggregate obtained in each preparation stage was studied using dynamic and electrophoretic light scattering. The initial measurements were conducted with colloids based on blank micelles. The study focused on evaluating the efficacy of the crosslinking process and he structural stability of the micelles. DLS analysis revealed the formation of relatively small particles by co-assembly of PEO_113_-*b*-PCL_35_-*b*-PEO_113_ and PAA_13_-*b*-PCL_35_-*b*-PAA_13_ in PBS, characterized with an average hydrodynamic diameter (D_h_) of 32 nm and zeta potential of −22 mV. These results are in a good agreement with those from our previous study performed in distilled water [27]. The negative zeta potential was mainly due to the presence of carboxyl groups. After crosslinking, the D_h_ of the micelles became smaller −29 nm (Figure 2A).

The zeta protentional of crosslinked micelles (−9 mV) is higher as compared to the non-crosslinked ones, which can be explained with the diminishing of free carboxyl groups in the micellar structure. The data are summarized in Table 1.

To prove the successful crosslinking reaction, the stability of CPMs against dissociation in organic solvent was evaluated. Hence, THF was substituted for PBS by dialyzing the aqueous colloid against the organic solvent. THF readily dissolves polymers, such as PCL, PAA, and PEO, as well as the copolymers on this basis. As expected, no particles were detected using DLS analysis when non-crosslinked micelles were placed in THF and stored at 25 °C for 24 h. In contrast, the measurement of CPMs, treated under similar conditions, revealed the existence of particles with nearly two times larger dimensions (D_h_ = 58 nm) and a broader particle size distribution (Figure 2B). Copolymer unimers were not identified in the sample. This swelling effect was probably due to the interaction of solvent molecules with the polymer segments out of the network (e.g., PCL, PEO). Nevertheless, the results from DLS in THF undoubtedly proved the good structural stability of CPMs, imparted by the crosslinking of PAA with cystamine. Incorporating S-S-groups causes the CPMs to develop a redox-responsive system, which is a key feature of nanocarriers designed for intracellular delivery of antitumor agents.

### 3.2. Drug Loading

**Figure 3:**
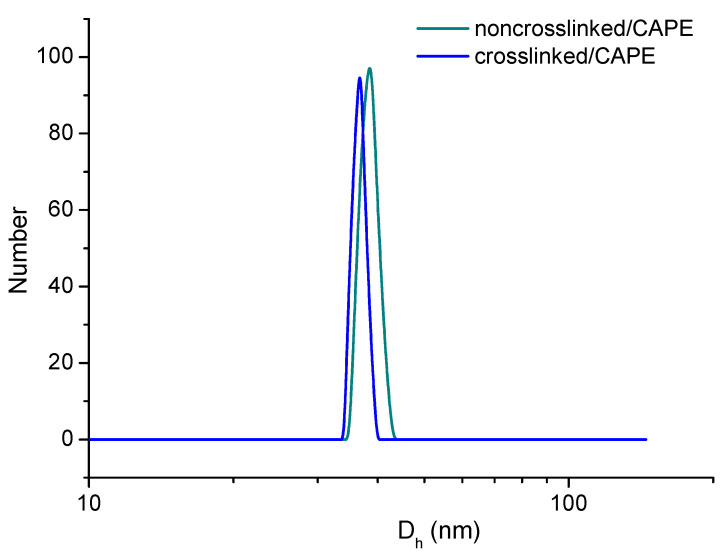
Size distribution plots of CAPE-loaded non-crosslinked and crosslinked micelles, based on PEO_113_-*b*-PCL_35_-*b*-PEO_113_ and PAA_13_-*b*-PCL_35_-*b*-PAA_13_ amphiphilic triblock copolymers, in PBS at 37 °C.

**Figure 4:**
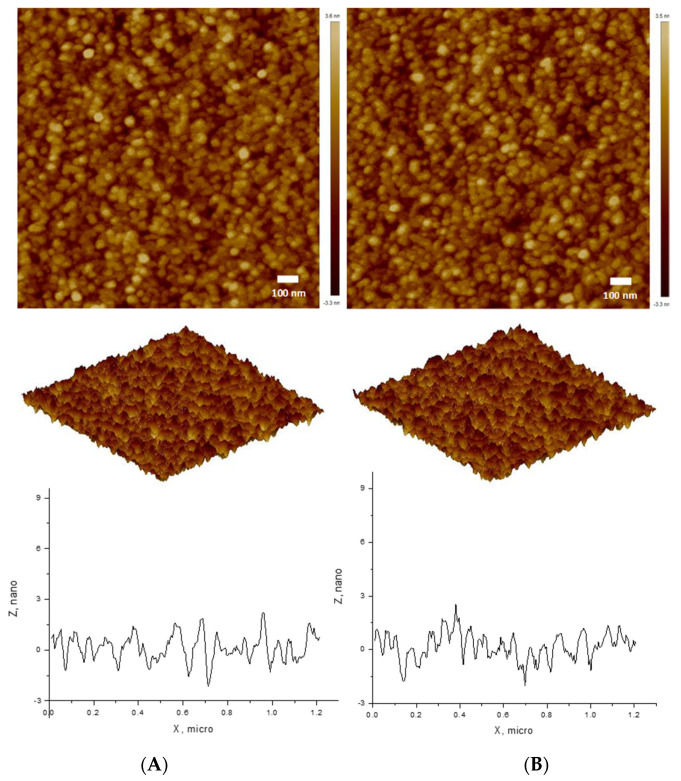
AFM tapping mode of 2D and 3D height images, and cross-section of (**A**) CAPE-loaded non-crosslinked micelles and (**B**) CAPE-loaded crosslinked micelles.

CAPE was entrapped into the PCL core of prepared non-crosslinked micelles via hydrophobic interactions. Then, the nanocarriers were stabilized by crosslinking with cystamine dichloride, as described above. The obtained CAPE-loaded carriers were characterized using DLS and ELS. The D_h_ of non-crosslinked drug-loaded mixed micelles was 38 nm, which is slightly higher compared to the size of blank micelles (Figure 3, Table 1). We suggest that the embedded CAPE increased the core size, and thus contributed to the overall growth of the micelles. After crosslinking, the diameter of drug-loaded micelles decreased to 36 nm due to slight shrinkage of the shell. We found that both the crosslinked and non-crosslinked systems were stable in PBS for a long time without visible agglomeration/precipitation. This means that the loading of CAPE into micellar carriers and the subsequent crosslinking did not interfere with the colloid stability of the system. Representative AFM micrographs of CAPE-loaded micelles, before and after crosslinking, are shown in Figure 4. Evidently, the micelles possess spherical shape and nanoscopic dimension. There is no significant difference in the carrier size and morphology between non-crosslinked and crosslinked micelles, which is consistent with DLS data. A standard procedure was followed to calculated an EE of 89.5% for the non-crosslinked micelles.

### 3.3. Drug Release

**Figure 5:**
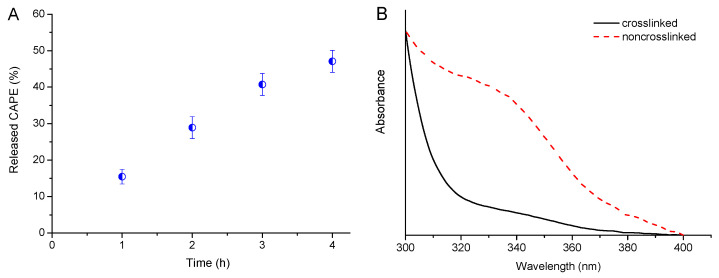
In vitro release of CAPE from non-crosslinked micelles (**A**), and UV-vis absorption spectra of samples taken from the external dialysis medium at the 4th hour of the release test for CAPE-loaded non-crosslinked and crosslinked micelles (**B**).

In vitro release of CAPE from the two types of carriers, non-crosslinked and crosslinked micelles, was initially evaluated at 37 °C in a phosphate buffer (pH 7.4) to mimic conditions in the bloodstream. Expectedly, the non-crosslinked micelles exhibited sustained release profile and approximately 45% of CAPE was released in 4 h (Figure 5A). In contrast, CPMs did not release a detectable amount of CAPE under the same conditions. Figure 5B demonstrates the obvious difference in the absorption spectra of the samples, taken from the external dialysis medium at the 4th hour of the experiments. Such a low absorbance at 330 nm for the CAPE-loaded CPMs was registered, even after 24 h. Definitely, the different performance of the micellar systems (sustained release vs. lack of release) is a result of the crosslinked middle layer of the CPMs. In other words, the crosslinking of a PAA blocks seems to be a valuable strategy for preventing premature leakage of a drug in the bloodstream.

**Figure 6:**
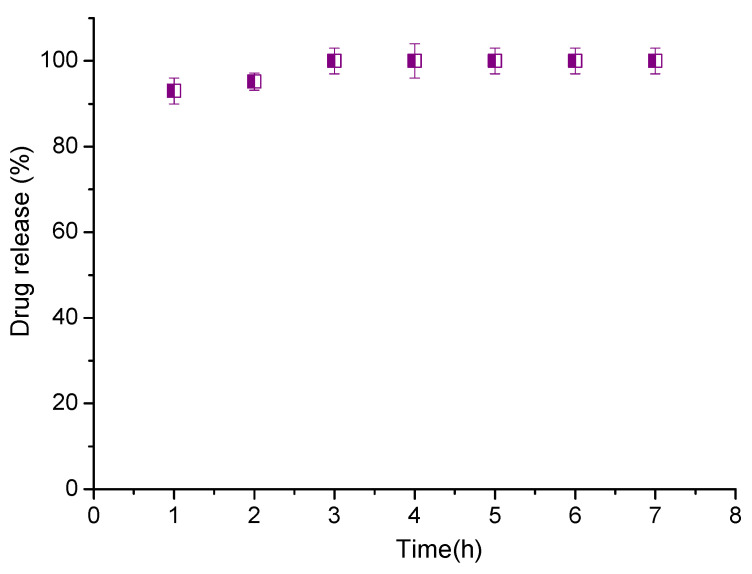
Simultaneous in vitro release of CAPE from crosslinked micelles in weak acidic medium pH = 5, after treatment with DTT.

The drug release behavior of the CAPE-loaded CPMs in weak acidic media, treated with a reducing agent, was assessed as well. The in vitro release test was carried out at pH 5.0 using the dialysis method. First, DTT (10 mM) was added to the colloid solution (pH 5) and the sample was allowed for 24 h to complete the de-crosslinking reaction. Then, the sample was placed in a dialysis tube and dialyzed against acidic buffer. The experiment was conducted under conditions chosen to imitative the harsh microenvironment in a tumor cell (e.g., in the late lysosomes), which favors the breakup of disulfide bonds. Indeed, nearly 93% of CAPE was released in 1 h (100% in 3 h), indicating that CLMs were successfully de-crosslinked in the reductive medium (Figure 6).

## 4. Discussion

The preparation and characterization of non-crosslinked multilayered micellar nanocarriers by co-assembly of PEO_113_-*b*-PCL_35_-*b*-PEO_113_ and PAA_13_-*b*-PCL_35_-*b*-PAA_13_ amphiphilic triblock copolymers was reported recently by our team [27]. The nanocarriers, comprising a PCL-core, a middle layer of PAA/PEO, and an outer PEO-corona layer, are designed to meet the basic requirements of nanomedicine. Hydrophobic PCL chains constructed the micellar core, intended for the solubilization of water-insoluble CAPE, and impart biodegradability to the carrier. We hypothesize that, due to the substantially different chain length of the hydrophilic segments (PEO >> PAA), a mixed PAA/PEO middle layer and an outer PEO-dominated corona layer were formed. The PEO corona serves for prolonged circulation of a carrier in the blood stream, as it can diminish the agglomeration of micelles and the adsorption of signaling proteins, responsible for the capture and elimination of carriers by the reticuloendothelial system [4]. The carboxyl groups of the PAA segments are suitable for complexation with positively charged molecules or a crosslinking reaction. It is noteworthy that the blank micelles used in the present study only caused a slight decrease in cell viability (up to 80%), which, according to the ISO 10993-5:2009 classification, is a low degree of toxicity [27].

CAPE was selected as a model anticancer drug to prove the concept for triggered drug release from the developed CPMs at a relatively high concentration of reducing agent, resembling intracellular tumor cell conditions. Very often, cancer treatments require intravenous administration of nanomedicine [1]. Depending on the tumor type, the injection site can be quite far from the tumor, so nanocarriers travel through the circulatory system for given amount of time (hours to days) before they can encounter a tumor. On this ground, CAPE-loaded CPMs designed for intracellular drug delivery were fabricated by co-assembly of the two block copolymers and crosslinking of the carboxyl groups of PAA segments with cystamine dihydrochloride. The efficiency of this reaction and the structural stability of the carrier, respectively, were confirmed using DLS analysis. The reason to present the DLS data as number-weighted plots is the light-scattering phenomenon discussed in detail in our early works [26,27]. In summary, we found that the micelles comprising PCL, constructed from either single copolymer or a copolymers blend, exhibited a bimodal particle size distribution in the intensity-weighted plot. For all samples studied, the dominant peak, associated with small micelles (D_h_~25–35 nm) was accompanied by a less intensive peak, attributed to larger aggregates/agglomerates (D_h_ > 100 nm). In volume- and number-weighted size distribution plots of the peak from the larger particles disappeared, and the peak from the micelles remained at the same position. These results, and the well-known phenomenon that larger particles scatter light much more intensely than smaller ones, are the arguments for considering polymer micelles as the main fraction of particles in the colloids. Therefore, for simplicity, DLS results from this study were given as number-weighted plots. Another important issue, proven in our early works, is that the co-assembly method can produce one population of mixed micelles rather than a mixture of single-polymer micelles [28]. In particular, mixed micelles based on PEO_113_-*b*-PCL_35_-*b*-PEO_113_ and PAA_13_-*b*-PCL_35_-*b*-PAA_13_ copolymers were obtained above a critical micelle concentration (*cmc*) of 0.078 gL^−1^ [27]. DLS/ELS measurements confirmed that single polymer micelles are not present in the sample with mixed micelles (see Table S1 in the Supplementary Information).

DLS and AFM studies revealed the formation of spherical nanosized mixed micelles. The small decrease in D_h_ after crosslinking is attributed to the slight shrinkage of the micelles because of the crosslinking of PAA segments located in the middle layer. The PAA network imparted superior stability against disaggregation of the structures and prevented premature drug release in a neutral media (pH 7.4, without reducing agent). In contrast, due to the labile SS-bond, a rapid and pronounced release of CAPE was found when CPMs were exposed to the action of the reducing agent (DTT). An incubation period of 24 h was chosen to ensure sufficient time for complete de-crosslinking of the micelles, although the protocol does not exactly mimic actual biological conditions. We believe that the slight delay in achieving a quantitative (100% in 3 h) release is probably due to hindered diffusion of CAPE across the membrane, and not to the interaction between the carrier and the drug molecules. The last finding confirmed the capability of CPMs to release CAPE, triggered by the reducing agent. In addition, considering the equal quantities of loaded and released CAPE, one can conclude that the crosslinking of the middle micellar layer has no effect on the drug encapsulation efficiency. The results from this study proved our concept, that the developed three-layered crosslinked micelles, comprising SS-bonds within the network, are redox-responsive systems with the potential for controllable release of CAPE in the tumor microenvironment.

## 5. Conclusions

Novel redox-responsive micellar nanocarriers for controllable release of caffeic acid phenethyl ester in tumor cells were developed by co-assembly of PEO_113_-*b*-PCL_35_-*b*-PEO_113_ and PAA_13_-*b*-PCL_35_-*b*-PAA_13_ and subsequent crosslinking. Prior to crosslinking, CAPE was entrapped in the hydrophobic PCL core via hydrophobic interactions. Purposely, crosslinking of poly(acrylic acid) segments using cystamine was accomplished in the middle layer of the three-layer micellar structures. As a result, the structural stability of the carriers was significantly improved, and the premature release of CAPE in neutral media (pH 7.4) was prevented, unlike for non-crosslinked micelles. On the other hand, the crosslinking of the middle layer contributed to the preservation of the high encapsulation efficiency of the system. Upon exposure of CPMs to the action of reducing agent (DTT), the SS-groups, incorporated into the polymer network, were cleaved, resulting in a rapid and pronounced release of CAPE. Based on the results obtained, we can consider the studied smart system a promising candidate for advanced cancer treatment.

## Figures and Tables

**Table 1:** Dynamic and electrophoretic light scattering data of blank and CAPE-loaded mixed copolymer micelles prepared from PEO_113_-*b*-PCL_35_-*b*-PEO_113_ and PAA_13_-*b*-PCL_35_-*b*-PAA_13_ amphiphilic triblock copolymers by co-assembly in PBS (pH 7.4).

Sample	D_h_ (nm)	ζ Potential (mV)
Non-crosslinked	32 ± 2	−22 ± 2
Crosslinked	29 ± 2	−9 ± 1
Non-crosslinked/CAPE	38 ± 3	−22 ± 2
Crosslinked/CAPE	36 ± 3	−9 ± 1

## Data Availability

Not applicable.

## Supplementary Materials

The following supporting information can be downloaded at: https://www.mdpi.com/article/10.3390/pharmaceutics14030679/s1, Table S1. Dynamic and electrophoretic light scattering data and critical micelle concentrations of single copolymer and mixed copolymer micelles.
