# Supplementary material for: Redox-Responsive Crosslinked Mixed Micelles for Controllable Release of Caffeic Acid Phenethyl Ester
Source: Pharmaceutics. 2022 Mar 20;14(3):679. doi: 10.3390/pharmaceutics14030679 (PMC8953340; doi:10.3390/pharmaceutics14030679)
Supplement: Supplementary file 1 [file pharmaceutics-14-00679-s001.zip › pharmaceutics-1638270-supplementary.pdf]

pharmaceutics

Supplementary Materials: Redox-Responsive Crosslinked Mixed Micelles for Controllable Release of Caffeic Acid Phenethyl Ester

DLS/ELS measurements revealed that single polymer micelles do not exist in the sample of mixed micelles. The determined diameter of mixed micelles (38 nm) is between D_h of the two single copolymer micelles (Table S1). The same tendency is found for zeta potential values (Table S1). The zeta potential of mixed micelles is -22 mV, while the single polymer micelles have zeta potential of −33 mV (PAA_{13}-*b*-PCL_{35}-*b*-PAA_{13}) and −0.6 mV (PEO_{113}-*b*-PCL_{35}-*b*-PEO_{113}), respectively. We have established that mixed micelles, comprising a nonionic and an ionic block, exhibit single zeta-potential value, unlike the mixture of two pre-formed single-polymer micelles (self-assembled separately and then mixed) [1]. Thus, our results are direct proof that only mixed micelles were formed from the PEO113-b-PCL35-b-PEO/PAA13-b-PCL35-b-PAA13 blend at the reported experimental conditions.

Table S1. Dynamic and electrophoretic light scattering data and critical micelle concentrations of single copolymer and mixed copolymer micelles.

**Copolymers**	**Hydrodynamic diameter (nm)**	**Zeta potential (mV)**	**Critical micelle concentration (g L^{-1})**
PAA_{13}-*b*-PCL_{35}-*b*-PAA_{13}	86 ± 3	−33 ± 3	0.063
PEO_{113}-*b*-PCL_{35}-*b*-PEO_{113}	26 ± 1	−0.6 ± 0.1	0.090
PEO_{113}-*b*-PCL_{35}-*b*-PEO_{113}/PAA_{13}-*b*-PCL_{35}-*b*-PAA_{13} *(molar ratio 3:1)*	38 ± 2	−22 ± 3	0.078

[1] Kamenova K, Haladjova E, Grancharov G, Kyulavska M, Tzankova V, Aluani D, et al. Co-assembly of block copolymers as a tool for developing novel micellar carriers of insulin for controlled drug delivery. *Eur Polym J.* **2018**, *104*, 1–9.
